# Single-Frame Vignetting Correction for Post-Stitched-Tile Imaging Using VISTAmap

**DOI:** 10.3390/nano15070563

**Published:** 2025-04-07

**Authors:** Anthony A. Fung, Ashley H. Fung, Zhi Li, Lingyan Shi

**Affiliations:** 1 Department of Biomedical Engineering, Yale University, New Haven, CT 06520, USA; 2 Department of Medicine, Yale School of Medicine, New Haven, CT 06520, USA; ashley.helmuth@yale.edu; 3 Shu Chien-Gene Lay Department of Bioengineering, University of California San Diego, La Jolla, CA 92093, USA; zhl026@ucsd.edu (Z.L.); l2shi@ucsd.edu (L.S.)

**Keywords:** vignetting, SRS nanoscopy, shading correction, tile stitching, image processing, artifact removal

## Abstract

Stimulated Raman Scattering (SRS) nanoscopy imaging offers unprecedented insights into tissue molecular architecture but often requires stitching multiple high-resolution tiles to capture large fields of view. This process is time-consuming and frequently introduces vignetting artifacts—grid-like intensity fluctuations that degrade image quality and hinder downstream quantitative analyses and processing such as super-resolution deconvolution. We present VIgnetted Stitched-Tile Adjustment using Morphological Adaptive Processing (VISTAmap), a simple tool that corrects these shading artifacts directly on the final stitched image. VISTAmap automatically detects the tile grid configuration by analyzing intensity frequency variations and then applies sequential morphological operations to homogenize the image. In contrast to conventional approaches that require increased tile overlap or pre-acquisition background sampling, VISTAmap offers a pragmatic, post-processing solution without the need for separate individual tile images. This work addresses pressing concerns by delivering a robust, efficient strategy for enhancing mosaic image uniformity in modern nanoscopy, where the smallest details make tremendous impacts.

## 1. Introduction

**Figure 1:**
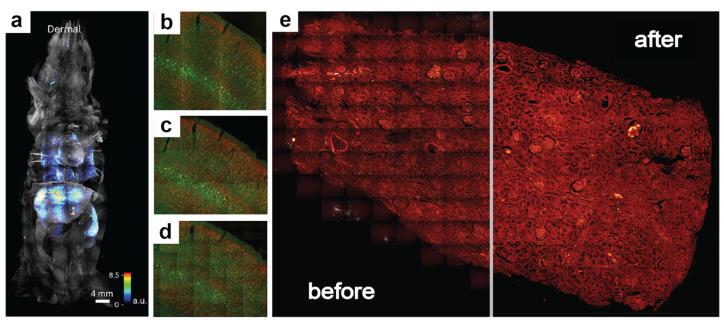
Examples of vignetting across different imaging modalities, and a demonstration of the results achieved by performing vignetting correction using VISTAmap. Shading artifacts can be seen in images obtained from various imaging modalities: light sheet (**a**), ribbon scanning confocal (**b**), confocal laser scanning (**c**), and swept field confocal (**d**) microscopy. Panel a scale bar is 4 mm, and panels b-d were imaged using 20× objectives. Panel (**e**) demonstrates the vignetting correction results obtained using VISTAmap. Figure 1a–d adapted with permission from Luo et al., 2025 [1] and Watson et al., 2017 [2] under the terms of the Creative Commons Attribution 4.0 International License.

Whether label-free or probed, imaging nanoscopy such as Stimulated Raman Scattering (SRS) or immunofluorescence involves powerful spatial multi-omics tools that rely on channel intensities to identify and analyze the molecular microenvironments of tissues. These techniques greatly benefit from large and diverse fields of view (FOVs), but to maintain high spatial resolution and image quality, they often entail a mosaic of stitched tiles. This is a consequence of the diffraction limit, which is a function of the numerical aperture (NA) of the objective lens. Higher NA lenses afford greater zoom and are typically associated with a higher signal-to-noise ratio (SNR) due to the focusing of the light source incident on the sample. They therefore offer finer pixel resolutions but suffer from smaller FOVs. If perfect infinite NA lenses and detectors existed, a whole slide image could be captured with extremely fine details and would only require one tile. However, due to physical limitations in lens geometry and detectable wavelengths at modest power, it is common to capture many small FOVs and stitch them together to afford both high resolution and the spatial context. Unfortunately, this often produces regularly interspersed shading artifacts, also referred to as vignetting artifacts, that appear like a grid of intensity fluctuations, especially if the FOV of each tile is large. Vignetting refers to the uneven intensity distributions near the borders of an image tile due to the shape of the lens and focal distance. It can be exacerbated by uneven focus planes, lensing and scattering effects, and poor beam alignment. These vignetting artifacts can cause considerable differences in super-resolution deconvolution, spectral unmixing, and other analyses. Given the wide range of technologies affected by vignetting artifacts, from traditional histological brightfield microscopy to fluorescence light sheet microscopy (Figure 1a–d) [1,2], there have been numerous efforts to correct them [3,4,5,6,7,8]. In the spirit of making image processing techniques more accessible, we present **VI**gnetted **S**titched-**T**ile **A**djustment using **M**orphological **A**daptive **P**rocessing (VISTAmap), a simple and readily accessible tool to correct for vignetting artifacts in post-stitched-tile grayscale images (Figure 1e).

Several conventional solutions to vignetting exist, such as increasing the overlap between tiles prior to acquisition, increasing the optical zoom, or capturing a background sample. However, these solutions require upstream effort and forethought, and increasing the tile overlap or zoom can cause significantly longer acquisition times. For a whole slide image (WSI) using a 20× objective lens, stitching patterns on the order of hundreds of tiles are common. Therefore, increasing the tile overlap from 5% to 10% for the same FOV can result in approximately 10% longer acquisition times, and increases in tandem with the number of tiles covered. Increasing optical zoom also mitigates vignetting but is often time/cost prohibitive for the same reasons. Downstream solutions such as pseudo flat-field background subtraction and frequency-based filtering generally work well but are heavily influenced by local pixel intensity distribution and range. Commercial software such as Huygens Stitcher, Olympus Fluoview, and Leica LASX can correct vignetting by sampling a representative background tile or selecting a default vignette profile to serve as the flat field subtractor. However, these methods operate on the individual tiles themselves prior to stitching, as do excellent tools such as MIST by Chalfoun and colleagues [3], CIDRE by Smith and colleagues [4], and BaSiC by Peng and colleagues [5]. VISTAmap differentiates itself from these tools by functioning on fully stitched images, not requiring the individual tiles as other methods do. This provides greater utility in cases where the individual tiles may not be readily available, such as when pulling images from online databases [9]. To give a general understanding of the various shading correction tools available, we have summarized a few with practical considerations in Table 1 below. In Table 1, the available tools are diverse in their method of operation but generally require individual tiles to function.

In this paper, we detail the VISTAmap workflow step-wise. We then demonstrate its performance on a whole slide SRS image and compare it with other common methods, such as background subtraction and band-pass filtering. We provide an example of how vignetting correction with VISTAmap can improve readouts by performing spatial deconvolution on SRS images before and after vignetting correction. To build on this, we use a computer-generated representation of vignetting to show how VISTAmap can improve feature detection.

## 2. Materials and Methods

The images used in this demonstration are SRS images that can be downloaded from the Shi Lab GitHub(version 1.0) https://github.com/lingyanshi2020/VISTAmap (accessed 06, April, 2025). Briefly, these images were acquired using a multi-photon microscope (Olympus FVMPE-RS) equipped with a 20× objective lens (NA 0.6). The pump–Stokes laser source (A.P.E picoEmerald, Berlin, Germany) with an average incident power of approximately 15 mW probes the Stimulated Raman Loss at 3010 cm^−1^, which is ascribed to the unsaturated fatty acid peak in the CH stretching region. The signal is captured with a PIN photodiode (Hamamatsu S3590-09, Hamamatsu City, japan) and digitized using a lock-in amplifier (Zurich Instruments HF2LI, Zurich, Switzerland). For hyperspectral image (HSI) stacks, the CH stretching region between 2750 cm^−1^ and 3150 cm^−1^ was subdivided into intervals of 5 wavenumbers and interpolated such that the interval is 1 wavenumber between data points to ensure consistency in plotting and analysis. All samples were fixed in 4% paraformaldehyde (PFA) and imaged in phosphate-buffered saline (PBS). Additional sample information can be found in the original publication [10].

Image analysis was conducted in a MATLAB R2024a live script which can be downloaded from the Shi Lab GitHub https://github.com/lingyanshi2020/VISTAmap (accessed 4 April 2025). The hardware and environment specifications are as follows:

- 13th Gen Intel(R) Core (TM) i9-13900H, 2600 MHz, 14 Core(s), 20 Logical Processor(s);
- 64 GB DDR4 RAM;
- Windows 11 Pro.

We did not approach any limits in processor or memory utilization and expect this tool to be used smoothly on a wide range of computers. Due to the simplicity of this method, memory usage and performance will be dominated by the size of the image being processed. Users may be able to decrease these limitations by down-sampling the image during the tissue detection and tile detection steps, and then up-sampling the resulting multiplier mask. The user cannot, however, split the image into sections for parallelization because the tile detection requires the entire image.

### 2.1. Loading the Image

**Figure 2:**
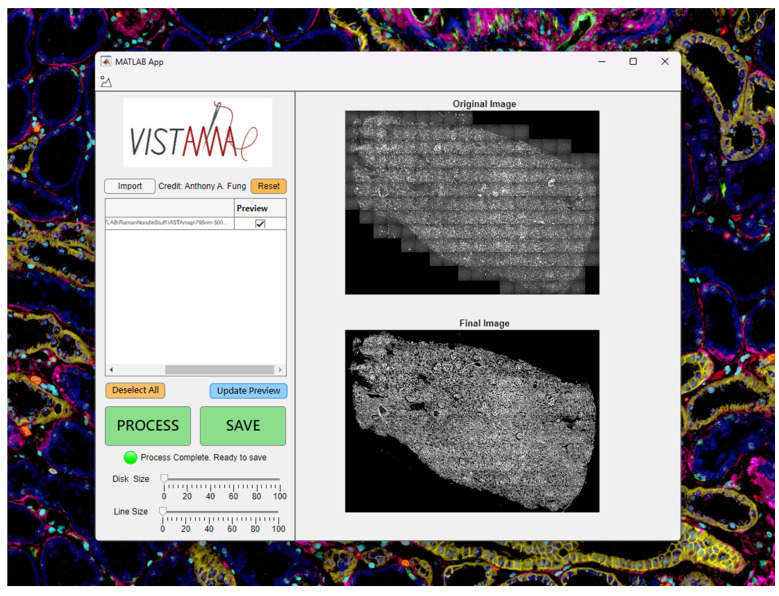
VISTAmap graphical user interface.

Users may import grayscale images in multiple formats such as TIFF (including ome.tiff and qptiff) and Olympus Bioformats (including oir and oib). Users may also import 2D MATLAB arrays (.mat) of any numerical type. Upon clicking the “import” button, a graphical user interface (GUI) will allow the user to select one or more image files, whose absolute path will be displayed in the UI table (Figure 2).

### 2.2. Deal with NaN and Inf Background

Several microscopy acquisition systems allow for uniquely drawn regions of interest (ROIs) that result in non-rectangular mosaics. In these cases, the empty space bound by the rectangular region and inscribed ROI may have a value of NaN or infinity (inf). These values can significantly impact the detection and processing of tissue. Therefore, NaN pixels are ignored, and inf pixels are set to the maximum saturated pixel value. The top 1% of pixel intensities (or all fully saturated pixels, whichever is greater in number) are temporarily withheld from the image processing due to their effect on the horizontal and vertical intensity profiles. These pixels are temporarily set to the average intensity of the tissue (excluding background) and will be reset to the original values at the end.

### 2.3. Isolating the Tissue

**Figure 3:**
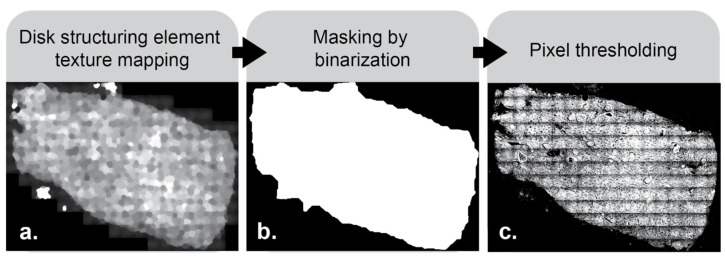
Texture filtering allows for content-aware background removal. A disk structuring element is applied, and identified textured regions can be visualized in panel (**a**). Binarization is performed on the pixels from the image in panel (**a**) using a set threshold. Pixels above the threshold are set to a value of 1 and boundaries are then smoothed (**b**). A mask is then created and overlaid on the original image to isolate the region of interest (**c**).

Non-rectangular mosaics have background pixels that skew the horizontal and vertical intensity profiles. Therefore, the tissue is isolated from the background using a disk structuring element to identify areas with texture (Figure 3a). Structuring elements are a common tool for use in image processing. They work by first centering the shape of choice, in this case a disk, on every pixel in the image. Morphological closing is first performed in two steps—dilation, then erosion. Dilation works by filling each disk with the maximum intensity value found within pixels beneath it. This expands bright areas and fills in dark gaps within the image. Erosion is then performed on the dilated image by filling each new disk with the minimum pixel intensity value from beneath the disk. This works to reduce the size of the created bright areas and bring them back closer to their original shape. Following closing, opening is performed. Opening works in the opposite order—erosion, then dilation. This accomplishes the inverse of closing by filling in bright areas within the image. The result of this morphological opening then closing process is a smoothed image, where intensity variations smaller than the chosen structuring element have been removed. Care needs to be taken in choosing the proper sizes for structuring elements. The code presented in this paper defaults to structuring element sizes tailored to use for a typical whole slide histological image, but they can be changed according to needs. The texture map created with the disk structuring element is then binarized based on a pixel intensity threshold (Figure 3b) to create an intermediate mask to identify only relevant pixels. These pixel values are thresholded appropriately (Figure 3c) and intersected with the original image to create a background-free image on which we operate to generate the horizontally and vertically averaged intensity profiles.

### 2.4. Identifying the Tile Boundaries

**Figure 4:**
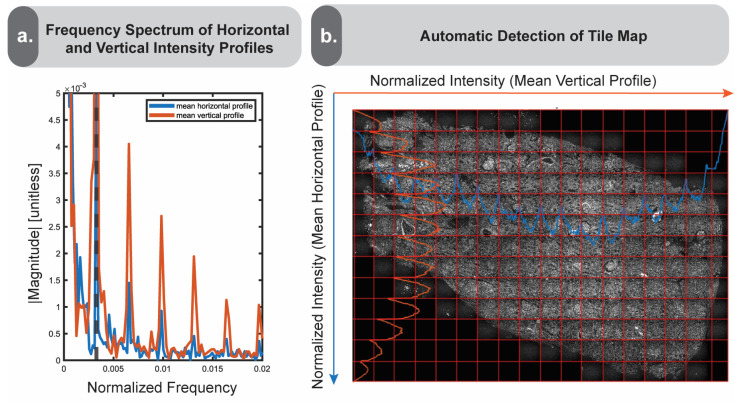
Frequency-based vignetted tile detection. Panel (**a**) depicts the calculated frequency spectrum of the image. The mean horizontal profile is displayed in blue, and the mean vertical profile in orange. The first major peak, marked by the dotted line, represents the frequency attributed to the vignetting patterns. The subsequent peaks may represent frequency variations attributed to smaller features withing the tissue. Panel (**b**) shows the average horizontal and vertical intensity profiles overlaid on the image, along with the automatically detected tile boundaries in red.

We transform the previously generated intensity profiles into the frequency domain by calculating the average pixel intensity by row and column, then using a Fast Fourier Transform (FFT) to output the frequencies. We then identify the first major peak of the output frequency spectrum, which is marked by a dotted line (Figure 4a). This first peak typically corresponds to the frequency of intensity variations associated with the image tiling. This offers a simple method to identify vignetting boundaries if the user is unaware of the real tile size a priori. We illustrate the result of this process in Figure 4b by overlaying the horizontally and vertically averaged intensity profiles on the original image, along with the frequency-derived tile grid estimations.

### 2.5. Extracting the Vignetting Effect

**Figure 5:**
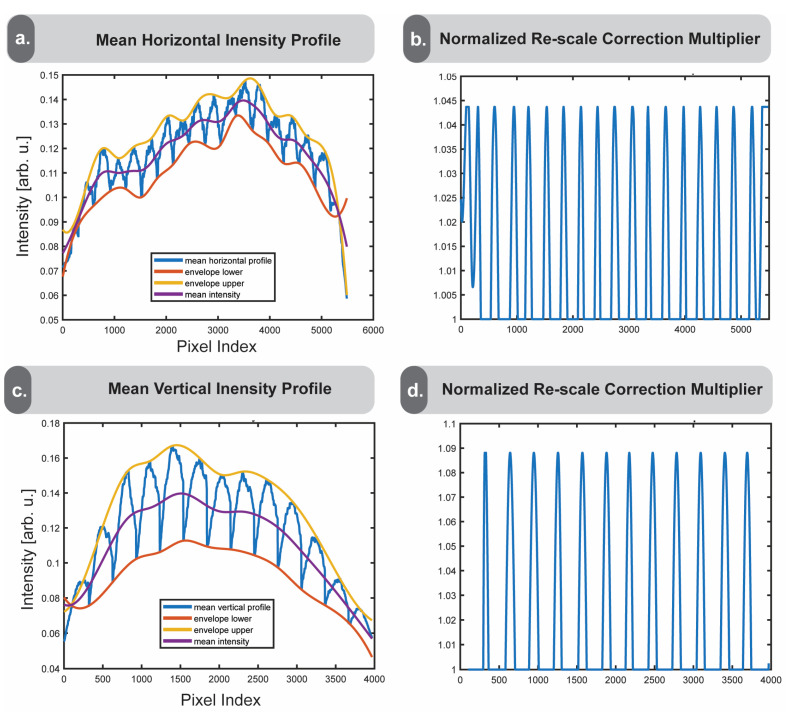
Determining the vignetting correction multiplier**.** Panels (**a**,**c**) show the horizontal and vertical mean intensity profiles in blue. The upper and lower bounds are shown in yellow and orange, respectively. The midline of the upper and lower bounds is drawn in purple. Panels (**b**,**d**) display the normalized horizontal and vertical correction multipliers, which can be applied to the image to reduce the horizontal and vertical intensity fluctuations.

The averaged intensity profiles are then enveloped by connecting the local minima and maxima with smoothed upper and lower lines, shown in yellow and orange, with the mean of the upper and lower bounds shown in purple (Figure 5a,b). An important feature of the enveloping function is that it accounts for the tile sizes detected in Section 2.4. Since the intensity profiles consist of not only the vignetting-associated frequency but the smaller tissue-feature-based frequencies as well, the tile size is needed to identify the appropriate minima and maxima. In cases where vignetting effects dominate, the purple midline represents a smoothed ideal case without vignetting. Dividing the midline by the troughs of their respective averaged intensity profiles returns a multiplier by which we can achieve a more smoothed image profile (Figure 5c,d). The reason we choose to apply this to the troughs of the intensity profile is because the vignette appears as a darker shaded area near the edges, while the properly focused regions are brighter. We constrain the peaks of the multiplier to the average peak height due to the assumption that the vignetting effect is consistent between tiles. These multipliers can then be applied to the image to correct the pixel intensities by the calculated amount.

### 2.6. Applying the Vignetting Correction

**Figure 6:**
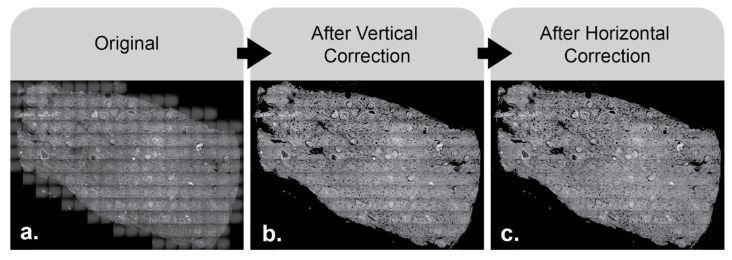
VISTAmap significantly reduces visible vignetting artifacts. The original image, before any processing, can be seen in panel (**a**). The image after vertical vignetting correction has been applied can be seen in panel (**b**). The image after horizontal and vertical vignetting correction has been applied can be seen in panel (**c**).

The results of the image after sequentially applying the horizontal and vertical multipliers are shown below (Figure 6).

### 2.7. Applying a Structuring Element for Finishing Touches

**Figure 7:**
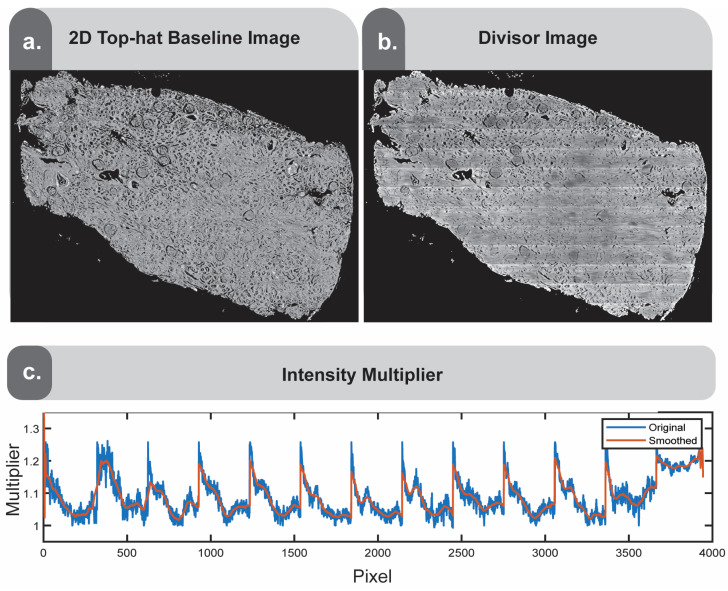
Morphological opening and closing operations using line structuring elements. (**a**) Masked disk-top-hat filtered image divided by a horizontal line structuring element-fitted image. (**b**) Result of the top-hat baseline image divided by the output of Figure 6. (**c**) The normalized vertical intensity profile of b, with a smoothed version using a moving average window of 51.

If the result is unsatisfactory, remaining finishing touches can be applied using morphological operations to generate a 2D top-hat baseline image (Figure 7a). Following the approach adapted by Leischner and colleagues [10], bright spots from the previously generated image that exceed a predefined brightness threshold are removed, and the resulting image is top-hat filtered using both a disk structuring element and line structuring element. Taking the ratio of these two images yields the baseline image (Figure 7a). This image represents a flat tissue image, preserving only the coarse morphologies. Since the goal is to modify the original image to approach this coarse flat image, we take the ratio of the baseline image and the image previously generated (Figure 6c) to obtain the divisor image (Figure 7b). From the resulting ratiometric image, we derive another whole slide intensity multiplier, which undergoes similar constraints and smoothing as described previously in Section 2.5 (Figure 7c). By smoothing and constraining this signal, we create a new multiplier image and apply it to the previous result image from Figure 6c. Due to the smoothing and constraints, it will approach the baseline image (Figure 7a) but will better preserve the finer details.

### 2.8. Re-Normalizing the Image to the Original Range

**Figure 8:**
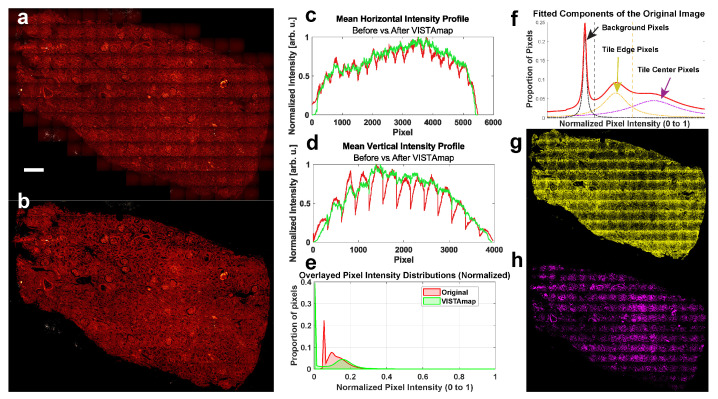
Evaluating the performance of VISTAmap. (**a**) The original whole slide SRS image of a human kidney necropsy at 2935 cm^−1^. Scale bar 500 microns. (**b**) VISTAmap output of a. (**c**,**d**) Horizontal and vertical, respectively, mean intensity profiles of a and b show that the predominant pixel intensity changes occur at the troughs of the vignetted boundaries, largely preserving the brightest, in-focus signal details. (**e**) Pixel intensity histograms of a and b show that the largest population of affected pixels are the background and vignetted regions. (**f**) Fitted peak distributions for the multimodal pixel intensity histogram of the original image. Vertical dashed lines threshold background pixels and vignetted pixels from the center tile pixels. The original pixel intensity distribution is shown in red, while the fitted peak component that is likely associated with the background is shown in black, that for edge pixels is shown in yellow, and that for center pixels is shown in purple. (**g**,**h**) Images displaying the corresponding thresholded pixels from the component traces in panel f confirm that the right-most front of the pixel distribution (purple component) corresponds to the pixels near the center of the image tiles.

The pixels in the final image are rescaled to the original range of the input image, and the top 1% of pixel intensities are restored. Before and after images are displayed (Figure 8a,b), along with their horizontally and vertically averaged intensity profiles (Figure 8c,d) and pixel intensity histograms (Figure 8e). The pixel intensity distribution did indeed change, which may preclude quantitative analysis. However, this is the intended effect, as most altered pixel intensities are the image background and shaded areas. The more intense areas (the right-most front of the distribution) are mostly unchanged. Several component peaks were fitted (Figure 8f) and used to threshold the pixels. The less intense bimodal peak in the original distribution (colored yellow) correspond mostly to the shaded pixels near the tile boundaries (Figure 8g), and one would expect these to be shifted into the more intense peak (colored magenta), which correspond mostly to the pixels near the centers of the tiles (Figure 8h).

## 3. Results

### 3.1. Demonstration of Issues with Flat Field Subtraction and Bandpass Filtering

**Figure 9:**
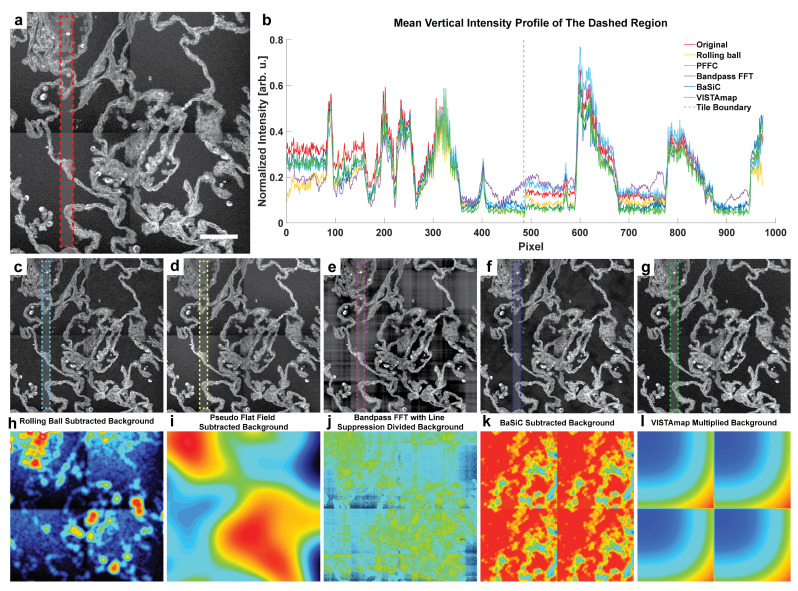
Comparison of VISTAmap performance and other methods. (**a**) Uncorrected human lung biopsy SRS image at 2935 cm^−1^. Scale bar is 100 microns. (**b**) Average intensity profile of the shaded regions of the SRS images. Tile boundary is marked by the vertical gray dashed line. (**c**) Rolling ball background subtraction result with a radius of 50 pixels. (**d**) Pseudo Flat Field Correction result from the Biovoxxel ImageJ plugin version 1.24.1 with a radius of 50 pixels. (**e**) Bandpass FFT result with horizontal and vertical line suppression with a low object threshold of 1 pixel and high object threshold of 200 pixels. (**f**) BaSiC result using automatic regularization parameters and ignoring dark field estimation. (**g**) VISTAmap result. Compared to results from other methods (**c**–**f**), VISTAmap results (**g**) show a considerably higher degree of vignetting correction and a lower degree of additional artifact formation. (**h**–**l**) Subtracted, divided, or multiplied flat fields from the respective methods of (**c**–**g**).

One issue with conventional methods such as pseudo flat field background subtraction and bandpass FFT for the removal of vignetting artifacts in stitched images is that it is difficult to differentiate background effects from tissue signals because these algorithms are generally not morphologically aware. That is, local pixel intensities from tissue interspersed with vignetted background can influence the flat field, especially when the signal is much more intense, yet sparser, than the background. Below is an example of a stitched laser-scanned SRS nanoscopy image of human lung tissue (Figure 9a). The mean vertical intensity profile for the dashed region of several post-processing techniques is displayed (Figure 9b). The lung’s wiry morphology contrasts with the background pixels in a sparse, weaving pattern, making it difficult to perform traditional background elimination through rolling ball background subtraction (Figure 9c), Pseudo Flat Field Correction (PFFC) [11] (Figure 9d), Bandpass FFT with line suppression (Figure 9e), and even BaSiC tile shading estimation by inexact augmented Lagrange multiplier robust sparse principal component analysis (RPCA) (Figure 9f). Average intensity profiles for these images show that aside from VISTAmap (Figure 9g), only the FFT method was able to eliminate the intensity shift at the tile boundary marked by the dashed gray line (Figure 9b). However, in the FFT method, there are several edge effects generated by local high-intensity pixels, resulting in even more horizontal and vertical shading artifacts. Respective background derivations for these methods show that even though the rolling ball method approached the flat field caused by the vignetting effect, the tissue morphology’s irregular texture became intertwined with the baseline estimation, which may cause spurious consequences after subtraction (Figure 9h–l).

### 3.2. Improved Spatial Deconvolution

**Figure 10:**
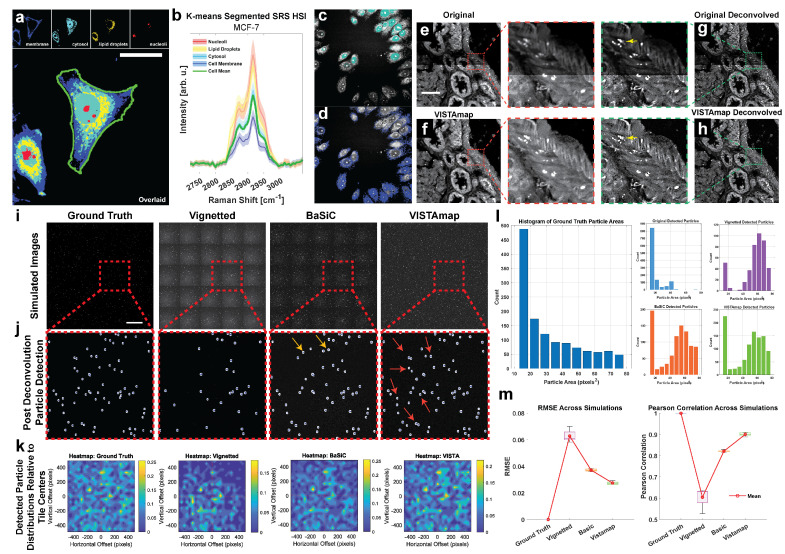
Vignetting effects on spectral and spatial resolution. (**a**) K-means clustering of an in vitro MCF-7 breast epithelial cell line with cell segmentation boundary highlighted in green. Scale bar is 10 microns. (**b**) Mean cluster spectra with 1 standard deviation error bands. (**c**) Phasor segmented pixels on in vitro MCF-7 breast cells show confounding clusters toward the bottom edge of the vignetted tile. (**e**,**f**) Before and after images of a human kidney biopsy region of interest near a tile boundary with zoomed regions in the dashed areas. Scale bar is 50 microns. (**g**,**h**) A-PoD deconvolved results of (**e**,**f**). Panels (**c**,**d**) are adapted with permission from Fung 2023 [10]. (**i**) Simulated stitched images of nanoparticles with added point spread function convolution, noise, and off-center vignetting. (**j**) Each simulated image was deconvolved and particles were detected. Scale bar is 20 microns. (**k**) Particle distribution is displayed as the density map of the average tile of each image. (**l**) Particle size distributions for each case show that vignetting effects significantly alter which particles were detected. (**m**) The RMSE and Pearson’s correlation coefficient were calculated for each case. This simulation was repeated 5 times. Box plots show the inner quartiles with the median line, and whiskers extend to the extreme non-outlier values (values beyond 3 median absolute deviations).

As a relatively new imaging platform in the biomedical space, SRS nanoscopy is concerned with two major development fronts: chemical resolution and spatial resolution. Increasing the signal-to-noise ratio and spectral resolution of a hyperspectral image (HSI), can have profound impacts on shortening acquisition times and enhancing image quality. Here, we demonstrate the negative impacts vignetting effects have on stitched hyperspectral images. At a subcellular level, pixel spectra of a HSI can be clustered into organelles such as nucleoli and lipid droplets (Figure 10a) even though the spectral shape of each cluster resembles the cell-average spectrum (Figure 10b). This is because SRS intensity is proportional to concentration and carries compartmental information in addition to multiplexed bond moieties. On the other hand, these differences in intensity can be confounded with other effects including vignetting near the boundaries. At a spectral level, several tools exist, such as The Unscrambler (Capterra), to correct for this using Multiplicative Scattering Correction (MSC) as a preprocessing normalization technique that favors the preservation of spectral shape over amplitude. To highlight this, we included demonstrations adapted from previous work that demonstrate phasor segmentation, which encodes amplitude and phase information, of an in vitro MCF-7 cell line culture. Toward the bottom edge, there is significant vignetting, which confounds two segmented regions (Figure 10c,d). This could be due to the presence of variable vignetting extents at each wavenumber in the HSI stack. That is, vignetting effects may manifest differently in each Raman peak, making it impossible to use the HSI stack as a whole to estimate a single flat field.

Spatially, deconvolution is often necessary to approach or exceed the diffraction limit, elucidating structures at the nanoscale. Here, we demonstrate single-frame deconvolution via Adam optimization-based Pointillism Deconvolution (A-PoD)—a super-position of virtual emitters-based method solved by gradient descent (Figure 10e,f) [12]. Local intensity differences, in addition to slight focus blur (depending on the dominant source of vignetting), can significantly impact the deconvolution result. For instance, lipid droplet structures are not resolved as well near the image tile boundaries (marked by yellow arrows) without vignetting correction (Figure 10g,h).

To further test this, a stitched image consisting of a grid of 5 × 5 tiles of 1024 × 1024 pixels each were generated. Then, 30–50 randomly sized particles with a diameter of 4–10 pixels (corresponding to approximately 40–100 nm particles) were randomly distributed. The intensity of the particles was also randomly generated but was multiplied by their diameters so that larger particles appeared brighter. Vignetting effects were simulated by convolving with a synthetic point spread function (PSF) (Gaussian radius of 21 pixels and sigma of 2), adding noise, and exponentially decaying the intensity from the center of the vignette according to the code in the supplementary materials (Figure 10i). After deconvolution, particles were detected (Figure 10j), and it is clear that the vignetted case had the fewest particles detected. Both BaSiC and VISTAmap were able to recover many of the missed particles, marked with arrows (Figure 10j). For each tile in the stitched images, the distribution of detected particles relative to the tile centers were averaged and displayed as density maps (Figure 10k). This confirms that the majority of detected particles were significantly reduced near the shaded margins of the vignetted case but were rescued by both BaSiC and VISTAmap processing. Smaller particles were particularly affected by vignetting (Figure 10l). Notably, BaSiC and VISTAmap recovered many of the smaller particles, with VISTAmap outperforming slightly. This simulation was repeated five times and the root mean square error (RMSE) and Pearson’s correlation coefficient (PCC) show that VISTAmap had the lowest error and highest correlation with the ground truth detected particle distribution (Figure 10m).

## 4. Discussion

Despite ongoing developments in optical setups and pre- and post-processing techniques, vignetting remains a prevalent issue in the field of nanoscopy. While many existing methods of vignetting correction may provide satisfactory results, most are reliant on making corrections in early stages of the image acquisition or stitching process. This proves limiting in cases where images have already been acquired, and significant time and resources would be needed to restart from an earlier stage in the process. Unlike other vignetting correction algorithms, such as BaSiC (which corrects for vignetting on individual tiles before stitching has taken place), VISTAmap can perform vignetting correction on whole stitched images. This simplifies workflows and allows correction on archived or collected images where individual tiles may no longer be available. VISTAmap leverages two simple concepts to afford this flexibility: morphological texture filters to delineate tissue from the background (allowing for the correction of non-rectangular stitching maps), and frequency-based tile detection (allowing for tailored enveloping of average intensity bands and ultimately the streamlined determination of the corrective multiplier). An interesting future work could combine VISTAmap’s automated tile detection to extract pseudo-tiles from post-stitched images for downstream use in other pipelines that require individual tiles such as BaSiC.

In nanoscopy applications, such as SRS, where image stitching is essential to study whole tissue dynamics at a nanoscale, VISTAmap shows particular promise. By eliminating the vignetting artifacts that would otherwise obscure key details, performance of segmentation, spectral unmixing, and super-resolution deconvolution algorithms can be improved. This allows for greater molecular insight to be retrieved from every sample.

While VISTAmap significantly minimizes vignetting effects and enhances image quality, it is essential to note that no vignetting correction tool is perfectly suited for all forms of quantitative analysis. Analyses that rely on absolute pixel intensities, such as some forms of quantitative spectral analyses, may be negatively impacted by the pixel intensity variations that occur due to vignetting. Vignetting correction algorithms like VISTAmap attempt to correct for these using intensity fluctuation patterns found throughout the image. However, since corrections are made from patterns and averages, they may not perfectly reflect the ground truth. This is not to say that VISTAmap cannot improve outcomes of quantitative measurements but rather that such measurements will not be directly based on the ground truth and should be interpreted with appropriate considerations. VISTAmap’s utility lies primarily in improving visual clarity for more qualitative analyses, such as molecular profiling, tissue segmentation, and spatial deconvolution. By removing artifacts, VISTAmap aids in these downstream analyses, which are critical in nanoscopy applications like hyperspectral molecular clustering and super-resolution imaging. Overall, VISTAmap offers a practical solution that enhances the usability of stitched nanoscopic images, ensuring that structural and molecular information can be extracted effectively from even the most vignetted datasets. It is our sincere hope that this tool will serve as an instructional resource for those interested in learning about image processing and the impacts vignetting artifacts may have in the SRS nanoscopy space.

## 5. Conclusions

VISTAmap is a simple tool for correcting shading artifacts in post-stitched images caused by vignetting. Its main advantages are tissue detection through morphological texture filtering and automated tile detection. This allows for the separation of tissue from the background in non-rectangular stitching maps of whole slide images and streamlined corrective multiplier mask generation due to informing the window size of the vignetting signal based on the detected tile size. We evaluated the performance of VISTAmap qualitatively in eliminating tile boundaries, and quantitatively in deconvolution and particle detection. Qualitatively, the tile boundaries in the stitched image were made less noticeable (Figure 9b) without generating defects as in the bandpass FFT and BaSiC methods (Figure 9e,f). Quantitatively, VISTAmap-corrected stitched images were able to detect more small nanoparticles (Figure 10l) and rescued the spatial distribution of detected particles from the vignetted simulation (Figure 10j,m) with a lower RMSE and higher correlation coefficient for the ground truth case than the BaSiC processed images. Although we compare this method to other simple techniques and advanced algorithms, we do not definitively claim VISTAmap to be better. This is because quantitative measures will depend highly on the analyte. For instance, both BaSiC and VISTAmap will perform better on larger grids of image tiles and are both sensitive to the nature of the image tiles (whether sparse particles or dense tissues). The analysis of image-dependent performance requires further exploration. The aim of this study is to introduce an open source tool, illustrate its core operations step-wise, and give a basic rationale and performance evaluation.

## Figures and Tables

**Table 1:** Selected vignetting artifact removal approaches listed alphabetically.

**Method**	**Year** **Published**	**Modus Operandi**	**Input Format**
BaSiC [5]	2017	Sparse robust decomposition and reweighted L1 norm minimization with smoothness constraints	Individual tiles for grayscale or color images (time lapse permitted)
CIDRE [4]	2015	Retrospective estimation and minimization of multiplicative and additive contributions away from “true” intensity distribution with smoothness constraints	Individual tiles for grayscale or color images (uncorrelated, no time lapse)
MIST [3]	2017	Global mosaic optimization via weighted maximum spanning tree (MST). Handles geometric corrections (Fourier-based) and illumination blending	Individual tiles for grayscale or color images (must have at least 10% tile overlap)
Principal Color Components [7]	2016	Low-pass filtering to determine shading, PCA to determine color components	Individual color image tiles, which can be irregularly mapped and shaped
Simple Shading Correction [6]	2020	Pixel intensity sorting and “smoothness” evaluation using local coefficient of variation (LCoV) to avoid artifacts	Individual color image tiles
SIMToolbox [8]	2020	Tile average intensity projection and border-limited Gaussian blurring	3D SIM image tiles (post-reconstruction of individual tiles)

## Data Availability

Code and demo images are available from the lab GitHub: https://github.com/lingyanshi2020/VISTAmap (accessed 4 April 2025).

## Supplementary Materials

The following supporting information can be downloaded at: https://www.mdpi.com/article/10.3390/nano15070563/s1.

## Abbreviations

The following abbreviations are used in this manuscript:

NA	Numerical aperture
SRS	Stimulated Raman Scattering
FOV	Field of view
SNR	Signal-to-noise ratio
A-PoD	Adam optimization-based Pointillism Deconvolution
PFFC	Pseudo Flat Field Correction
HSI	Hyperspectral image
PBS	Phosphate-buffered saline
PFA	Paraformaldehyde
GUI	Graphical user interface
PSF	Point spread function
ROI	Region of interest
FFT	Fast Fourier Transform
RSPCA	Robust sparse principal component analysis
MSC	Multiplicative Scatter Correction
VISTAmap	VIgnetted Stitched-Tile Adjustment using Morphological Adaptive Processing
