# Supplementary material for: Single-Frame Vignetting Correction for Post-Stitched-Tile Imaging Using VISTAmap
Source: Nanomaterials (Basel). 2025 Apr 7;15(7):563. doi: 10.3390/nano15070563 (PMC11990085; doi:10.3390/nano15070563)
Supplement: Supplementary file 1 [file nanomaterials-15-00563-s001.zip › nanomaterials-3552351-supplementary.pdf]

```
%% Simulation and Analysis Over Multiple Iterations
numIterations = 5;    % Run the entire simulation 5 times
numDatasets = 4;      % Conditions: Ground Truth, Vignetted, VISTA, BaSiC

% Preallocate arrays to store RMSE and Pearson correlation for each dataset
RMSE_all = zeros(numIterations, numDatasets);
Corr_all = zeros(numIterations, numDatasets);

% Define constant simulation parameters:
tileSize = 1024;      % Each tile is 1024x1024 pixels
numTiles = 5;         % 5x5 grid of tiles
overlap = 50;         % 50-pixel overlap between adjacent tiles
shift = tileSize - overlap;  % Shift for stitching

% PSF parameters for simulation (same for all iterations)
psfSize  = 21;
psfSigma = 2;
PSF = fspecial('gaussian', psfSize, psfSigma);

% Define the number of bins and histogram edges for the density maps
numHistBins = 25;
edgesX = linspace(-tileSize/2, tileSize/2, numHistBins+1);
edgesY = linspace(-tileSize/2, tileSize/2, numHistBins+1);

% Precompute tile centers in the stitched image (same for all iterations)
tileCenters = zeros(numTiles*numTiles, 2);  % each row: [row_center, col_center]
idx = 0;
for i = 1:numTiles
    for j = 1:numTiles
        idx = idx + 1;
        tileCenters(idx, :) = [ (i-1)*shift + tileSize/2, (j-1)*shift + tileSize/2 ];
    end
end

% Names of the dataset conditions (order must match below)
datasetNames = {'Ground Truth','Vignetted','VISTA','BaSiC'};

for iter = 1:numIterations
    fprintf('Running simulation iteration %d\n', iter);

    %% Cell 1: Generate Ground Truth Tiles and Stitch Them Together
    % Preallocate cell array to store ground truth tiles
    groundTiles = cell(numTiles, numTiles);
    allParticleAreas = [];   % store areas (not used further in RMSE, but kept for
completeness)

    % Particle parameters
    minParticles = 40;
    maxParticles = 60;

    for row = 1:numTiles
        for col = 1:numTiles
```

```
            img = zeros(tileSize, tileSize);
            numParticles = randi([minParticles, maxParticles]);
            for p = 1:numParticles
                % Particle radius in [2,5] biased toward smaller sizes.
                r = 2 + (5 - 2) * (rand()^2);
                particleArea = pi * r^2;
                allParticleAreas = [allParticleAreas; particleArea]; %#ok<AGROW>

                margin = ceil(r) + 1;
                cx = randi([margin, tileSize - margin]);
                cy = randi([margin, tileSize - margin]);
                intensity = 0.3 + 0.05 * rand() + 0.1 * ((r - 2) / (5 - 2));
                [X, Y] = meshgrid(1:tileSize, 1:tileSize);
                mask = ((X - cx).^2 + (Y - cy).^2) <= r^2;
                img(mask) = img(mask) + intensity;
            end
            % Smooth the tile to simulate spherical particles.
            imgSmooth = imgaussfilt(img, 1.5);
            groundTiles{row, col} = imgSmooth;
        end
    end

    % Stitch ground truth tiles together.
    stitchedHeight = shift * (numTiles - 1) + tileSize;
    stitchedWidth  = shift * (numTiles - 1) + tileSize;
    stitchedImage = zeros(stitchedHeight, stitchedWidth);
    for row = 1:numTiles
        for col = 1:numTiles
            rStart = (row - 1) * shift + 1;
            cStart = (col - 1) * shift + 1;
            stitchedImage(rStart:rStart+tileSize-1,      cStart:cStart+tileSize-1)      =
groundTiles{row, col};
        end
    end

    %% Cell 2: Create Vignetted Simulation Dataset
    % Process ground truth tiles to simulate microscope acquisition.
    % Preallocate cell arrays for vignetted and VISTA map tiles.
    vignetteTiles = cell(numTiles, numTiles);

    % Pre-calculate vignette mask and blur weight (same for all tiles)
    [X, Y] = meshgrid(1:tileSize, 1:tileSize);
    refPoint = [tileSize/3, 2*tileSize/3];  % [row, col]
    D = sqrt((Y - refPoint(2)).^2 + (X - refPoint(1)).^2);
    D_norm = (D - min(D(:))) / (max(D(:)) - min(D(:)));
    k = 2;
    vignetteMask = exp(-k * D_norm);
    blurWeight = D_norm;  % 0 for bright (sigma=1) and 1 for dark (sigma=3)

    for row = 1:numTiles
        for col = 1:numTiles
            tile = groundTiles{row, col};
```

```
            tileConv = imfilter(tile, PSF, 'conv', 'same');
            tileBlur1 = imgaussfilt(tileConv, 1);
            tileBlur3 = imgaussfilt(tileConv, 3);
            tileBlur = (1 - blurWeight) .* tileBlur1 + blurWeight .* tileBlur3;
            noiseStd = 0.01;
            tileNoisy = tileBlur + noiseStd * randn(size(tileBlur));
            tileFinal = tileNoisy .* vignetteMask;
            vignetteTiles{row, col} = tileFinal;
        end
    end

    % Stitch the vignetted tiles together.
    stitchedVig = zeros(stitchedHeight, stitchedWidth);
    for row = 1:numTiles
        for col = 1:numTiles
            rStart = (row - 1) * shift + 1;
            cStart = (col - 1) * shift + 1;
            stitchedVig(rStart:rStart+tileSize-1,       cStart:cStart+tileSize-1)       =
vignetteTiles{row, col};
        end
    end


    %% Cell 3: Apply BaSiC Correction to Vignetted Tiles and Re-Stitch
    % Combine vignetted tiles into a 3D array (adding a small offset to avoid zeros)
    numTilesTotal = numTiles * numTiles;
    IF_v = zeros(tileSize, tileSize, numTilesTotal);
    idx = 1;
    offsetVal = 1e-3;
    for i = 1:numTiles
        for j = 1:numTiles
            tile = im2double(vignetteTiles{i,j});
            IF_v(:,:,idx) = tile + offsetVal;
            idx = idx + 1;
        end
    end
    [flatfield, ~] = BaSiC(IF_v, 'darkfield', 'false');

    % Apply flatfield correction to each vignetted tile.
    correctedTiles = cell(numTiles, numTiles);
    for i = 1:numTiles
        for j = 1:numTiles
            tile = im2double(vignetteTiles{i,j});
            correctedTiles{i,j} = tile ./ flatfield;
        end
    end

    % Stitch the BaSiC-corrected tiles.
    stitchedbasicImage = zeros(stitchedHeight, stitchedWidth);
    weightImage = zeros(stitchedHeight, stitchedWidth);
    for i = 1:numTiles
        for j = 1:numTiles
```

```
            row_start = (i-1)*(tileSize-overlap) + 1;
            col_start = (j-1)*(tileSize-overlap) + 1;
            row_end = row_start + tileSize - 1;
            col_end = col_start + tileSize - 1;
            stitchedbasicImage(row_start:row_end, col_start:col_end) = ...
                stitchedbasicImage(row_start:row_end,        col_start:col_end)        +
correctedTiles{i,j};
            weightImage(row_start:row_end, col_start:col_end) = ...
                weightImage(row_start:row_end, col_start:col_end) + 1;
        end
    end
    stitchedbasicImage = stitchedbasicImage ./ weightImage;

    %% Cell 4: Compare 2D Particle Density Maps using RMSE and Pearson Correlation
    % Define the dataset images for analysis.
    datasetImages = {stitchedImage, stitchedVig, stitchedvistamap, stitchedbasicImage};

    % Parameters for deconvolution and particle detection.
    deconvIter = 20;
    radiusRange = [2 5];

    % Initialize a cell array to hold heatmaps for each condition.
    heatmaps = cell(1, numDatasets);

    % Loop over each dataset.
    for d = 1:numDatasets
        currentImage = im2double(datasetImages{d});
        deconvImage = deconvlucy(currentImage, PSF, deconvIter);

        % Set imfindcircles parameters conditionally.
        if strcmp(datasetNames{d}, 'Ground Truth')
            [centers, ~, ~] = imfindcircles(deconvImage, radiusRange, ...
                'ObjectPolarity', 'bright', 'Sensitivity', 0.99);
        elseif strcmp(datasetNames{d}, 'Vignetted')
            deconvImage = imgaussfilt(deconvImage, 1);  % slight smoothing
            [centers, ~, ~] = imfindcircles(deconvImage, radiusRange, ...
                'ObjectPolarity', 'bright', 'Sensitivity', 0.9, 'EdgeThreshold', 0.1);
        else
            [centers, ~, ~] = imfindcircles(deconvImage, radiusRange, ...
                'ObjectPolarity', 'bright', 'Sensitivity', 0.9);
        end

        % Compute offsets of each detected particle relative to the nearest tile center.
        numParticles = size(centers, 1);
        offsets = zeros(numParticles, 2);
        for k = 1:numParticles
            detectedCol = centers(k, 1);
            detectedRow = centers(k, 2);
            dists  =  sqrt((tileCenters(:,1)  -  detectedRow).^2  +  (tileCenters(:,2)  -
detectedCol).^2);
            [~, minIdx] = min(dists);
            assignedCenter = tileCenters(minIdx, :);
```

```
            offsets(k,   :)   =   [detectedRow   -   assignedCenter(1),   detectedCol   -
assignedCenter(2)];
        end
        offsets(:,1) = -offsets(:,1);  % flip vertical offset if needed

        % Create a 2D histogram (density map) of offsets.
        [counts, ~, ~] = histcounts2(offsets(:,2), offsets(:,1), edgesX, edgesY);
        heatmap = counts / (numTiles*numTiles);
        heatmaps{d} = heatmap;
    end

    % Compute RMSE and Pearson correlation of each heatmap versus Ground Truth.
    GT_heatmap = heatmaps{1};
    rmseVals = zeros(1, numDatasets);
    corrVals = zeros(1, numDatasets);
    for d = 1:numDatasets
        diffMap = heatmaps{d} - GT_heatmap;
        rmseVals(d) = sqrt(mean(diffMap(:).^2));
        corrVals(d) = corr2(heatmaps{d}, GT_heatmap);
    end

    % Store the metrics for this iteration.
    RMSE_all(iter, :) = rmseVals;
    Corr_all(iter, :) = corrVals;

    fprintf('Iteration %d metrics:\n', iter);
    for d = 1:numDatasets
        fprintf('%s:  RMSE  =  %.4f,  Corr  =  %.4f\n',  datasetNames{d},  rmseVals(d),
corrVals(d));
    end
end

%% Plot RMSE and Pearson Correlation Box Charts in a Tiled Layout with Custom Colors and
Mean Line
% Reorder the metrics so that the order is:
% 1: Ground Truth, 2: Vignetted, 3: Basic (BaSiC), 4: Vistamap.
RMSE_reordered = RMSE_all(:, [1, 2, 4, 3]);
Corr_reordered = Corr_all(:, [1, 2, 4, 3]);

% Define the dataset names in the desired order.
datasetNames_ordered = {'Ground Truth','Vignetted','Basic','Vistamap'};

% Define desired colors for each group.
colorsFill = {[0.68, 0.85, 0.9], [0.8, 0.7, 0.8], [1, 0.8, 0.6], [0.7, 1, 0.7]};
colorsEdge = {[0, 0, 1], [1, 0, 1], [1, 0.55, 0], [0, 1, 0]};

% Number of groups and iterations.
numGroups = 4;
numIter = numIterations;  % already defined from simulation loop

% Create a tiled layout with 1 row and 2 columns.
figure;
```

```
t = tiledlayout(1,2, 'TileSpacing','Compact','Padding','Compact');

% First tile: RMSE Boxplots with Custom Colors
nexttile;
hold on;
% For each group, plot its boxchart at the corresponding x position.
for i = 1:numGroups
    % x positions for this group
    x = repmat(i, numIter, 1);
    boxchart(x, RMSE_reordered(:, i), ...
        'BoxFaceColor', colorsFill{i}, 'BoxEdgeColor', colorsEdge{i}, 'MarkerStyle',
'none');
end
% Set the x-axis ticks and labels.
set(gca, 'XTick', 1:numGroups, 'XTickLabel', datasetNames_ordered);
ylabel('RMSE');
title('RMSE Across Simulations');
% Calculate group means and overlay a red line.
groupMeansRMSE = mean(RMSE_reordered, 1);
plot(1:numGroups, groupMeansRMSE, '-o', 'LineWidth', 2, 'Color', 'r');
hold off;

%Second tile: Pearson Correlation Boxplots with Custom Colors
nexttile;
hold on;
for i = 1:numGroups
    x = repmat(i, numIter, 1);
    boxchart(x, Corr_reordered(:, i), ...
        'BoxFaceColor', colorsFill{i}, 'BoxEdgeColor', colorsEdge{i}, 'MarkerStyle',
'none');
end
set(gca, 'XTick', 1:numGroups, 'XTickLabel', datasetNames_ordered);
ylabel('Pearson Correlation');
title('Pearson Correlation Across Simulations');
groupMeansCorr = mean(Corr_reordered, 1);
plot(1:numGroups, groupMeansCorr, '-o', 'LineWidth', 2, 'Color', 'r');
hold off;
```
